# Debris and Smear Layer Removal in Curved Root Canals: A Comparative Study of Ultrasonic and Sonic Irrigant Activation Techniques

**DOI:** 10.3390/dj12030051

**Published:** 2024-02-26

**Authors:** Ronald Wigler, Yara Srour, Yuval Wilchfort, Zvi Metzger, Anda Kfir

**Affiliations:** 1 Department of Endodontology, The Goldschleger School of Dental Medicine, Tel Aviv University, P.O. Box 39040, Tel Aviv 6997801, Israel; metzger@post.tau.ac.il (Z.M.); andak@post.tau.ac.il (A.K.); 2 Private Clinic, Eilabun 1697200, Israel; dryarasrour@gmail.com; 3 The Goldschleger School of Dental Medicine, Tel Aviv University, P.O. Box 39040, Tel Aviv 6997801, Israel; yuval.wilchfort@gmail.com

**Keywords:** debris, smear layer, sonic activation, ultrasonic activation

## Abstract

The aim of this study was to compare the cleaning efficacy of three irrigant activation devices with a control of non-activated syringe and needle irrigation in curved root canals. Sixty human curved roots were endodontically prepared and divided into four groups (n = 15) with similar root curvature distributions. Final irrigation using 4% NaOCl was performed with a syringe and needle (30-G) alone, or with Eddy sonic powered irrigation system (polymeric tip #0.25/0.06), Endosonic ultrasonic activation (polymeric tip #0.25/0.03), or Irrisafe ultrasonic activation (stainless-steel tip, #0.25/0.00). SEM was used to evaluate cleaning efficacy, employing five-score systems for debris and smear layer. While no significant difference in debris removal was observed between Endosonic or Irrisafe activation and non-activated irrigation, Eddy sonic powered irrigation system significantly improved debris removal in the apical third of curved root canals. Smear layer removal was effective in coronal and mid-root sections for all groups but less so in the apical third. Thus, Eddy sonic powered irrigation system demonstrated higher efficacy in removing debris from the apical third of curved root canals compared with non-activated syringe and needle irrigation. However, all three irrigant activation systems exhibited no difference from the non-activated control in smear layer removal.

## 1. Introduction

The successful outcome of root canal treatments significantly depends on the meticulous cleaning and shaping of the root canal system [1]. Achieving optimal root canal cleanliness involves the complete removal of infected debris [2,3]. However, this task presents a substantial challenge due to the intricate nature of the root canal system and the limitations of mechanical instrumentation techniques [4,5]. Moreover, mechanical instrumentation generates a smear layer, which not only hampers disinfection but also presents a significant hurdle for thorough cleaning as it penetrates dentinal tubules and adheres to root canal walls [2,6].

Studies have shown that approximately 10–80% of root canal walls remain untouched by mechanical instruments [7,8,9,10]. This underscores the importance of adjunct chemical methods for cleaning [11]. Consequently, various techniques and irrigant delivery devices have been proposed to enhance the effectiveness of chemical cleaning and disinfection following mechanical instrumentation [3,12,13].

The current state of dental knowledge does not indicate a single optimal root canal irrigation protocol for achieving clinical success in endodontic treatment. There are various irrigants available, including NaOCl, EDTA, chlorhexidine gluconate, citric acid, distilled water, and saline, as well as numerous techniques for their activation. While literature suggests an advantage to irrigant activation compared with non-activated irrigation, no single method is recommended as superior to others [14]. The choice of technique heavily depends on the specific anatomy of the patient’s root canal and their predisposition. A recent systematic review and meta-analysis by Kumar et al. [14] comparing the efficacy of different irrigant activation techniques for irrigant penetration depth into the root canal system showed that irrigation activation techniques significantly improved irrigant delivery up to the WL in both straight and curved root canals compared with the conventional syringe and needle irrigation technique. Therefore, the researchers recommend incorporating recent irrigant activation techniques into routine endodontic practice [14].

A systematic review of 15 in vitro studies by Nagendrababu et al. [15] concluded that the use of irrigant activation can lead to superior microbial reduction within the root canal system.

A recent systematic review and meta-analysis by Virdee et al. [16] assessed whether irrigant activation techniques improve smear layer and debris removal compared with conventional non-activated syringe and needle irrigation and found that all activation techniques enhanced intracanal cleanliness, although none achieved the complete removal of debris and smear layer from the root canal walls. The apical part of the root canal, with its complex anatomical spaces such as apical deltas, isthmuses, and lateral canals, poses a particular challenge for effective cleaning, especially in curved root canals [17,18].

Passive ultrasonic irrigation (PUI) utilizing a stainless-steel non-cutting wire tip is extensively cited in the literature [11,12,13,14,15,16,17,18,19,20,21,22,23,24]. It employs cavitation and acoustic microstreaming to enhance irrigant cleaning efficiency [25,26]. However, its efficacy in curved root canals is limited due to the instrument’s restricted oscillation freedom [27,28,29]. Moreover, the engagement of the metal tip with the walls of the curved root canals may unintentionally result in the removal of small amounts of dentin while activating the irrigant [22,24]. Such uncontrolled dentin removal has the potential to contribute to the development of an undesirable smear layer [22,24]. An alternative option that might overcome this limitation is sonic activation with a polymer tip [30]. Eddy sonic powered irrigation system (EDDY, VDW, Munich, Germany) is an airscaler-powered activation system featuring a flexible polyamide tip operating at a frequency of 5000 to 6000 Hz. According to the manufacturer (VDW), the tip moves in an oscillating motion with a high amplitude, leading to three-dimensional movement that induces cavitation and acoustic streaming, associated with enhanced cleaning efficiency. A recent systematic review and meta-analysis by Chu et al. [31] demonstrated that sonic activation utilizing a polymer tip (EDDY, VDW) was at least equivalent to PUI concerning smear layer and debris removal and might serve as a substitute.

EndoSonic PS (SelectD, Seoul, Republic of Korea) is an ultrasonic-powered irrigant activation system that utilizes an autoclavable tip made of flexible polyether ketone polymer and is designed, according to the manufacturer, to work in both straight and curved root canals, allowing the safe removal of debris and smear layer from the root canal system (SelectD). In instances of breakage, the manufacturer asserts that the fractured component can be readily removed through irrigation (SelectD). EndoSonic PS (SelectD) was shown to be as effective as PUI with an Irrisafe ultrasonic activation tip (Satelec Acteon Merignac-Cedex, France)) and sonic activation with Eddy sonic powered irrigation system (VDW) regarding smear layer removal in straight root canals [24]. However, there are no published data on the efficacy of EndoSonic PS (SelectD) in curved root canals.

The present study aimed to evaluate and compare the efficacy of EndoSonic PS (SelectD), Eddy sonic powered irrigation system (VDW), Irrisafe ultrasonic activation (Satelec Acteon), and non-activated syringe and needle irrigation in removing debris and smear layer in curved root canals with that of syringe and needle irrigation without additional activation. The null hypothesis posits no difference in smear layer and debris removal among the three irrigant activation systems and non-activated syringe and needle irrigation.

## 2. Materials and Methods

The study design was based on the methodologies outlined by Mancini et al. [32], Haupt et al. [21], and Wigler et al. [24] and received approval from the Institutional Ethics Committee (IEC No. 55.19).

### 2.1. Tooth Selection and Preparation

**Figure 1:**
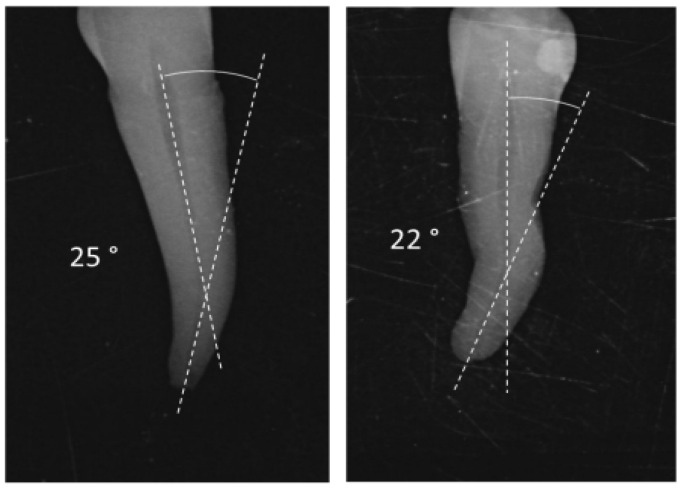
Measuring root canal curvature by Schneider’s method.

A total of 60 intact human mandibular premolars with curved roots were selected from recently extracted teeth, which had been extracted due to periodontal reasons. The teeth were mounted on a Protrain endodontic system (Simit Dental, Mantova, Italy) and radiographically evaluated using bucco-lingual and mesio-distal projections to verify the presence of a single root canal. The radiographic evaluation followed the method described by Iqbal et al. [33], and the angle of curvature of each root canal was determined according to Schneider’s method [34]. Teeth with root canals having an angle of curvature ranging from 20° to 30° were included in this study (Figure 1). This range was chosen to mirror common clinical scenarios and challenges encountered in curved root canals. Teeth not meeting these specified criteria were excluded from this study.

A coronal access cavity was created using diamond burs (Komet Dental, Lemgo, Germany), and apical patency was confirmed using a no. 10 K file (Mani, Utsunomiya, Japan). The working length (WL) was determined by observing the point at which the file first emerged through the apical foramen under magnification (Carl Zeiss Meditac, Dublin, CA, USA) and deducting 1 mm from this length. A glide path was prepared using a no. 15 K-file (Mani). To maintain consistency, a standardized 16 mm WL was established for all teeth by carefully cutting and grinding the crowns using a diamond disc (Henry Schein, Inc., Melville, NY, USA). Only teeth with intact root apices and a root canal width near the terminus compatible with a no. 15 K-file were included [35]. The apices were covered with wax (Baseplate wax, St. George Technology, Wilmington, NC, USA) to guarantee a closed root canal system.

All root canals were prepared by a single experienced operator (Y.S.) up to size 40/.04 using 2Shape instruments (MICRO-MEGA, Besançon cedex, France). Following each instrumentation, the root canals were irrigated with 1 mL of 4% sodium hypochlorite (NaOCl) solution using a syringe and a 30-G needle (NaviTip; Ultradent, South Jordan, UT, USA). At the end of the preparation, the root canals were rinsed with 5 mL of 17% ethylenediaminetetraacetic acid (EDTA). The roots were then assigned to four groups (n = 15), ensuring a similar distribution of root canal curvatures among the groups. These groups were then randomly assigned, using research randomizer software (Social Psychology Network, Wesleyan University, Middletown, CT, USA), to three experimental groups and one control group. Final irrigation was performed with a total volume of 6 mL of 4% NaOCl and was activated in the experimental groups using one of the tested activation protocols detailed below.

### 2.2. Final Irrigant Activation Protocols

Eddy sonic powered irrigation system (VDW) was performed with a 25/.06 polymer tip powered by an airscaler handpiece (W&H, Bürmoos, Austria) set to 6000 Hz. Endosonic PS (SelectD) PUI was performed with a 25/.03 polymer tip, and Irrisafe (Satelec Acteon) PUI was performed with a 25/.00 stainless-steel, non-cutting tip, both powered by an ultrasonic system (Satalec P5, Satelec Acteon) set at 30% (approximately 30,000 Hz). In all activation groups, the tip was inserted 1 mm from the WL, and three 20-s activation cycles were carried out, utilizing 2 mL of NaOCl in each cycle.

In the control group, no activation was performed; the root canals were irrigated with 6 mL of 4% NaOCl.

In all groups, irrigation solutions were delivered using a syringe and a 30-G needle (NaviTip; Ultradent), inserted 1 mm from the WL without binding. Subsequently, all root canals were irrigated with 5 mL of distilled water and dried with sterile paper points (MICRO-MEGA). (Table 1).

### 2.3. Scanning Electron Microscope (SEM) Analysis

The roots were longitudinally split to evaluate the remaining debris and smear layer. To prevent contamination by dentine particles during splitting, a gutta percha cone size 40/.04 (MICRO-MEGA) was passively placed in the root canals. Using a diamond disc (Horico, Berlin, Germany) under copious water irrigation, the external surface of each root was longitudinally grooved along the outer side of the curvature. Care was taken to avoid penetration into the root canal. The root was then longitudinally split into two halves using a chisel (Hu-Friedy, Chicago, IL, USA).

For each root, the half containing the most visible part of the root canal wall was preserved and coded. Horizontal marks were made on the fractured root surface with a scalpel (Hu-Friedy), indicating the center of the coronal, mid-root, and apical thirds of the root canal. This ensured the unbiased selection of areas for examination during scanning electron microscope (SEM) analysis.

The teeth were fixed in glutaraldehyde, dehydrated in a graded series of ethanol solutions, coated with gold, and observed under an SEM (JSM-25S, Jeol, Tokyo, Japan). Photomicrographs at ×200 and ×1000 magnifications were captured from the center of each third of the root canal. The selection of areas for imaging was determined using objective parameters: the line connecting the scalpel marks, indicating the center of the root segments, and the midpoint of this line. All images were coded to avoid bias when evaluating debris and smear layer removal effectiveness in each root canal third.

Two calibrated observers (R.W. and A.K.) independently performed a blind evaluation of the SEM images using a 5-score index system for debris and smear layer presence on the root canal surface. A total of 360 images were coded, and each image was analyzed twice at a 4-week interval. The observers were unaware of the image identities.

Debris was characterized as remnants of pulp tissue, dentine chips, and particles loosely adhering to the walls of the root canal [36]. The smear layer was described as a surface film comprising dentin particles, remnants of pulp tissue, and bacterial elements that persisted on dentine after root canal instrumentation [36]. Each image was assessed on a scale from 1 to 5 for the presence of debris/smear layer, utilizing the rating system introduced by Hulsman et al. [36], which has been applied in previous studies [24,37].

The criteria for debris assessment were defined as follows: Score 1: Root canal walls were observed to be clean with only a few particles present. Score 2: Small accumulations were noted, covering less than 25% of the root canal wall. Score 3: Accumulations covered 25–50% of the root canal wall. Score 4: Accumulations covered 50–75% of the root canal wall. Score 5: Accumulations covered more than 75% of the root canal wall with debris.

In evaluating the smear layer, the following criteria were applied: Score 1: Dentin tubules were found to be open; no smear layer was detected. Score 2: Some dentin tubules were open, with a minor amount of smear layer. Score 3: Few dentin tubules were open, and a consistent smear layer was observed along almost the entire root canal wall. Score 4: No open dentin tubules were visible; the root canal wall was completely covered with a consistent smear layer. Score 5: A thick, uniform smear layer covered the entire root canal wall. All scoring procedures were individually conducted by each examiner. In cases of scoring disagreement, discussions were held, and a consensus was reached.

**Figure 2:**
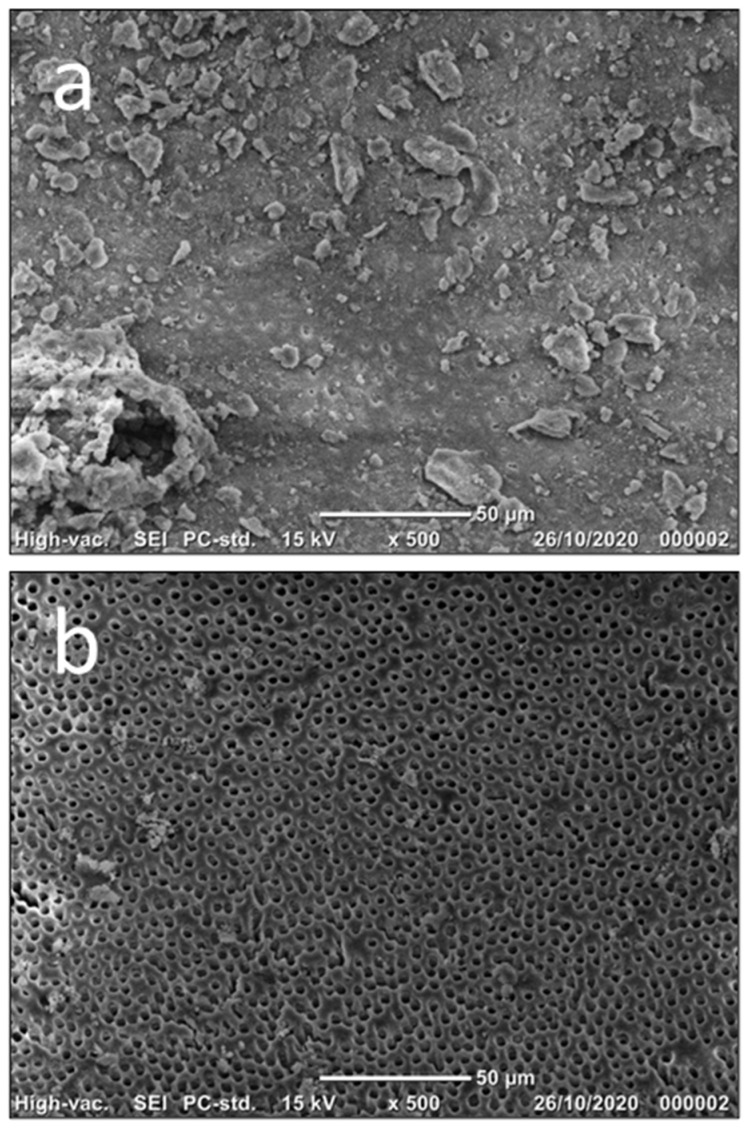
Illustrative images of “not clean” (**a**) and “clean” (**b**).

The scores for both debris and smear layer were initially recorded on a scale of 1–5. Subsequently, these scores were dichotomized into two groups: “Clean”, comprising scores 1 and 2, and “Not clean,” encompassing scores 3, 4, and 5. Illustrative images are presented in Figure 2.

### 2.4. Statistical Analysis

SAS 9.4 program (SAS Institute, Cary, NC, USA) was used to analyze the data. Multinomial mixed models with repeated measures for each tooth were used to assess differences between segments of the root canals. Multinomial models were used to assess differences between the thirds of the root canals. The significance level was set at *p* ≤ 0.05. Intra- and inter-examiner reliability were verified by the Kappa test. A sample size of 15 teeth per group ensures an 85% statistical power at a 5% significance level, allowing the detection of an 8% difference between the irrigation methods.

## 3. Results

The inter- and intra-observer agreement, as indicated by kappa values, was 0.867 and 0.901, respectively.

**Debris Removal**: In the coronal third of the root canal, all groups achieved a “clean” status, ranging from 87% to 93% of the cases, with no significant differences observed between the groups (Table 2). In the mid-root third, debris removal was slightly less effective, with “clean” root canals found in 67% to 80% of cases across all groups, showing no notable disparities. However, in the apical third, the Eddy (VDW) group exhibited complete cleanliness (100%), while the no-activation control group only achieved a “clean” status in 53% of cases, indicating a significant difference (*p* < 0.03). The disparity in debris removal between the coronal and apical thirds was particularly evident in the non-activated group, where the apical third was less effectively cleaned (53% “clean”) compared with the coronal third (93% “clean”, *p* < 0.041). There were no significant differences between the coronal and apical thirds in the Endosonic PS (Select D) and Irrisafe (Satelec Acteon) groups. Debris removal in the apical thirds in the latter two groups did not differ from that of the non-activated group (Table 3). The null hypothesis was rejected for debris removal.

**Smear Layer Removal**: In the coronal third of the root canal, all protocols effectively removed the smear layer, with 100% of cases displaying a “clean” root canal wall, and no significant differences were observed between the four groups (Table 4). In the mid-root third, smear layer removal ranged from 67% to 80% of cases achieving a “clean” status, with no notable distinctions among the groups. In the apical third, none of the protocols managed to adequately remove the smear layer, with 40% to 53% of cases showing a “not-clean” root canal wall (Table 5). There were no significant differences between the groups in this regard. Furthermore, significant differences were noted between the coronal and apical thirds in smear layer removal for all four groups (*p* < 0.013, *p* < 0.001, *p* < 0.003, and *p* < 0.0001 for the Eddy (VDW), Endosonic PS (SelectD), Irrisafe (Satelec Acteon), and no-activation groups, respectively). Accordingly, the null hypothesis was accepted for smear layer removal.

## 4. Discussion

In cases of infected root canals, the principal aim of endodontic treatment is to eradicate the debris and smear layer, thus effectively eliminating a substantial portion of microorganisms [1,2,3]. Recognizing the limitations of mechanical instrumentation alone in achieving thorough root canal cleaning, the incorporation of additional irrigation with antimicrobial agents and the activation of the irrigant become essential steps for comprehensive disinfection of the entire root canal system [3,4,5,7,8,9,10].

The primary aim of this study was to assess the cleaning efficacy of three different activation protocols for final irrigation in curved root canals, comparing them with non-activated irrigation, which served as the control. The findings revealed that all three protocols effectively removed debris from the coronal and mid-root thirds of the root canal. However, in the apical third, Eddy sonic powered irrigation system (VDW) demonstrated superior efficacy, significantly outperforming the non-activated control (*p* < 0.03). In contrast, the Endosonic PS (SelectD) and Irrisafe (Satelec Acteon) ultrasonic activators did not exhibit any significant advantage over the non-activated control in debris removal from any root canal region.

The differential performance observed in the apical third can be attributed to the unique mode of action of the Eddy sonic powered irrigation system. Sonic energy produces one single node and antinode over the entire length of the vibrated object, resulting in tip amplitudes and movements that remain virtually unaffected by contact with dentinal walls [13,38]. Unlike ultrasonic tips, which may face limitations in curved root canals due to file-wall contact, the sonic activator, operating at lower frequency but with higher displacement amplitudes, maintains effectiveness even when in contact with dentinal walls [19,20,27,39]. This characteristic likely contributed to its superior performance in the apical third of curved root canals, where other methods fell short.

Regarding smear layer removal, all experimental protocols, including the non-activated control, showed high and comparable efficacy in the coronal and mid-root thirds of the root canal. However, in the apical third, none of the protocols demonstrated significant superiority, leaving smear layer residues on root canal walls in 40% to 53% of cases. This finding aligns with the ongoing debate in the literature regarding the most effective activation technique, especially in curved root canals. While numerous studies have compared various activation methods with syringe and needle irrigation, indicating their overall superiority [10,11,12,13,14,15,16,17,18,19,20,21,22,23], the complexities introduced by canal curvature appear to impact not only conventional syringe and needle irrigation but also the added cleaning effect of irrigant activation. In curved and constricted canals, even the smallest needles will fall short of reaching the apical third, thus affecting irrigation efficacy. The present results emphasize that in the case of curved root canals, the benefits of activating the final irrigant might be even less pronounced.

In a guide presented in 2022 by the British Endodontic Society [39], it is emphasized that the primary objective of endodontic treatment is the elimination of microorganisms from the root canal system and preventing reinfection. The aims of the irrigation are defined as antibacterial action, tissue-dissolving capabilities, reduction in the friction between the instrument and the dentine, improvement of the cutting effectiveness of the files, lowering of the operation field’s temperature, and a washing effect to flush out the debris. Most importantly, irrigation is the only way to impact areas of the root canal wall untouched by mechanical instrumentation. Activation of irrigants by different methods is also recommended by the British Endodontic Society [40]. In a review by Mozo et al. [41], based on many in vitro studies, it is emphasized that activation of irrigants in general and ultrasonic irrigation in particular have a positive effect on the chemical, biological, and physical debridement of the root canal system. This review, which analyzed articles available on MEDLINE and Cochrane databases, concluded that in order to adequately compare different methods of activation of root canal irrigants, there is a need for protocols’ standardization, which is not yet available [40].

Recent systematic reviews have shown that ultrasonic activation is generally more effective than syringe and needle irrigation in removing pulp tissue remnants and hard tissue debris [16,21]. However, most of the included studies focused on straight root canals. Our results, in the context of curved root canals, indicate that the added cleaning effect of the activation of the final irrigant might be less pronounced.

The efficacy of irrigation is significantly influenced by factors such as apical preparation size, taper, needle gauge, and insertion depth [42,43,44,45]. Khademi et al. [33] demonstrated that a minimum preparation to size #30 is essential for proper penetration of irrigants into the apical third of the root canal. Studies have indicated that apical enlargement beyond size 35/.04 improves smear layer removal at the apical third using syringe and needle irrigation [46]. The optimal size for apical preparation remains a subject of debate, with larger apical preparations associated with an increased risk of iatrogenic errors, including root canal transportation, zipping, or perforation [47]. In line with Haupt et al. [23], preparation up to size 40/.04 was undertaken in this study to achieve a satisfactory balance between apical enlargement, cleaning efficacy, and the likelihood of procedural errors. The absence of significant differences in smear layer removal between the non-activated irrigant and all three activation protocols in the apical third can be attributed to the specific parameters employed in this study, specifically preparation to size 40/.04 using nickel–titanium rotary instruments and irrigation with a 30-G needle inserted up to 1 mm from the WL. The size and the taper of the root canal preparation, coupled with the use of a thin needle for precise delivery of both NaOCl and EDTA solutions, likely facilitated effective irrigation even without activation.

The influence of activation on the removal of the debris and smear layer in curved canals has been examined in only a limited number of studies [23,28,48,49].

Some of these studies have shown a benefit in additional activation [23,28,48], while others showed no difference [46]. Discrepancies among these studies can be attributed to variations in the degree of the root canal curvature, apical preparation size and taper, the gauge of the irrigation needle, insertion depth, irrigants used, their concentrations, the volume of the irrigant delivered, and the duration of activation [23,28,48,49]. Additionally, differences in the size of activation tips and files used may have contributed to the variations observed. Therefore, further studies should consider the diverse shaping parameters and activation protocols specific to curved root canals to provide a more comprehensive understanding of their impact on cleaning efficacy.

## 5. Conclusions

The coronal and mid-root thirds of the curved root canals were effectively cleaned with or without final irrigant activation, while cleaning the apical third remained a challenge. Debris was effectively removed from the apical third by the Eddy sonic powered irrigation system but not by the other methods of activation. However, none of the activation systems can effectively remove all the smear layer from the apical third of the curved root canals.

## Figures and Tables

**Table 1:** Sonic and ultrasonic Irrigant activation devices.

Device	Manufacturer	Handpiece	Tip	Frequency
Eddy Sonic Activation	VDW	Airscaler handpiece (W&H)	25/.06 polymer tip	6000 Hz
Endosonic PS	SelectD	Satalec P5, (Satelec Acteon)	25/.03 polymer tip	30,000 HZ
Irrisafe	Satelec Acteon	Satalec P5, (Satelec Acteon)	25/.00 Stainless-steel tip	30,000 Hz

**Table 2:** Presence of debris after each of the irrigant activation protocols. Dichotomized data: Scores 1 and 2: “Clean”. Scores 3, 4, and 5: “Not Clean”.

	Coronal Third		Mid-Root Third		Apical Third	
	Clean	Not Clean	Clean	Not Clean	Clean	Not Clean
Eddy	14 (93%)	1 (7%)	10 (67%)	5 (33%)	15 (100%)	0
Endosonic	13 (87%)	2 (13%)	11 (73%)	4 (27%)	11 (73%)	4 (27%)
Irrisafe	14 (93%)	1 (7%)	11 (73%)	4 (27%)	11 (73%)	4 (27%)
No Activation	14 (93%)	1 (7%)	12 (80%)	3 (20%)	8 (53%)	7 (47%)

**Table 3:** Differences in remaining debris between irrigation/activation methods in the apical third of curved root canals (*p* values).

	Eddy	Endosonic	Irrisafe	No Activation
Eddy	-	NS	NS	<0.03
Endosonic	NS	-	NS	NS
Irrisafe	NS	NS	-	NS
No activation	<0.03	NS	NS	-

**Table 4:** Presence of smear layer after each of the irrigant activation protocols. Dichotomized data: Scores 1 and 2: “Clean”. Scores 3, 4, and 5: “Not Clean”.

	Coronal Third		Mid-Root Third		Apical Third	
	Clean	Not Clean	Clean	Not Clean	Clean	Not Clean
Eddy	15 (100%)	0	12 (80%)	3 (20%)	9 (60%)	6 (40%)
Endosonic	15 (100%)	0	12 (80%)	3 (20%)	7 (47%)	8 (53%)
Irrisafe	15 (100%)	0	10 (67%)	5 (33%)	8 (53%)	7 (47%)
No Activation	15 (100%)	0	11 (73%)	4 (27%)	7 (47%)	8 (53%)

**Table 5:** Differences in remaining smear layer between irrigation/activation methods in the apical third of curved root canals (*p* values).

	Eddy	Endosonic	Irrisafe	No Activation
Eddy	-	NS	NS	NS
Endosonic	NS	-	NS	NS
Irrisafe	NS	NS	-	NS
No activation	NS	NS	NS	-

## Data Availability

The original contributions presented in the study are included in the article, further inquiries can be directed to the corresponding author.
